# Overexpression of TUBA1A suppresses bladder cancer progression with potential modulation of autophagic activity and macrophage polarization

**DOI:** 10.3389/fimmu.2026.1921855

**Published:** 2026-10-21

**Authors:** Ding-ming Song, Hong Lu, Yi-chang Shu, Jia-ju Xu, Yi-bo He, He Duan, Yan-yang Jin, Shi-liang Chen, Shi-chao Duan, Guan-hua Jing, Jia-ming Wen, Ming Tong

**Affiliations:** 1 Department of Urology, Zhejiang University, The Second Affiliated Hospital of Zhejiang University, Hangzhou, Zhejiang, China; 2 Department of Urology, Jinzhou Medical University, The First Affiliated Hospital of Jinzhou Medical University, Jinzhou, Liaoning, China; 3 Department of Congenital Heart Disease, General Hospital of Northern Theater Command, Postgraduate Training Base of Jinzhou Medical University, Shenyang, Liaoning, China; 4 Department of Clinical Lab, The First Affiliated Hospital of Zhejiang Chinese Medical University (Zhejiang Provincial Hospital of Chinese Medicine), Hangzhou, Zhejiang, China; 5 Henan Provincial People’s Hospital, Zhengzhou University People’s Hospital, Zhengzhou, Henan, China; 6 Department of Urology, Henan Provincial People’s Hospital, Zhengzhou University People’s Hospital, Zhengzhou, Henan, China

**Keywords:** aggrephagy, bladder cancer, immune microenvironment, single-cell RNA sequencing, TUBA1A

## Abstract

**Background:**

Bladder urothelial carcinoma (BUC) exhibits high recurrence and poor prognosis, with limited efficacy of current immunotherapies. The role of the aggrephagy-related gene TUBA1A in the BUC immune microenvironment remains unclear.

**Methods:**

Single-cell transcriptomic data from public repositories were analyzed to characterize the immune microenvironment. Non-negative matrix factorization identified aggrephagy-related immune–stromal subtypes, and CellChat mapped intercellular interactions. TIDE validation and KEGG enrichment defined functional modules. TUBA1A expression and function were evaluated through qRT-PCR, Western blot, proliferation, migration, apoptosis assays, and transmission electron microscopy. THP-1-derived macrophages were treated with conditioned medium from control or TUBA1A-overexpressing bladder cancer cells in the presence or absence of the autophagy inhibitor chloroquine (CQ), and polarization markers were assessed by flow cytometry and qRT-PCR. *In vivo* xenograft models verified tumor growth effects.

**Results:**

Single-cell analysis identified a TUBA1A-high macrophage subset with strong immune communication and immune activity. TUBA1A was significantly downregulated in BUC tissues and cell lines. TUBA1A overexpression inhibited proliferation, migration, and invasion, promoted apoptosis, and enhanced autophagic activity (increased LC3-II and ATG5, reduced SQSTM1/p62, and elevated autophagic vesicles). Conditioned medium from TUBA1A-overexpressing cells promoted M1-like polarization; CQ abolished the induction of the M1 markers IL-12 and CD86. *In vivo*, TUBA1A overexpression significantly suppressed xenograft tumor growth and reduced Ki-67 expression.

**Conclusion:**

TUBA1A is downregulated in BUC and acts as a candidate regulator of autophagic activity and macrophage polarization. TUBA1A overexpression is associated with inhibition of BUC progression, and its promotion of M1-like macrophage polarization appears to require autophagic activity. TUBA1A may represent a promising biomarker and therapeutic target for BUC.

## 1 Introduction

Bladder Urothelial Carcinoma (BUC) is a common malignant tumor of the urinary tract, ranking 10th among male cancers worldwide, with increasing incidence and mortality rates (1), especially in China where aging is advancing and pollution is severe (2, 3). Treatment methods include surgery, chemotherapy, and radiotherapy (4), but high recurrence (50%-70%) (5) and metastasis limit the therapeutic effect and affect patients’ quality of life (4, 6). The efficacy of immune checkpoint inhibitors is limited due to individual differences and rapid development of drug resistance (5, 7). The dynamic mechanisms of the tumor immune microenvironment remain poorly understood, which hinders targeted therapy (1, 8), underscoring the need to explore pathogenic mechanisms and immune networks (9, 10).

Autophagy degrades damaged components to maintain homeostasis (11). Aggrephagy is a selective form of autophagy that targets protein aggregates for lysosomal degradation while (12), and it can clear misfolded proteins (13, 14). Autophagy has dual roles in tumors (15), and aggrephagy in tumors has attracted attention. This study explores the expression of aggrephagy-related genes in the immune microenvironment of BUC, their interactions with immune components, and the mechanisms of tumor progression through combined analysis of single-cell and bulk transcriptomes, supplemented by extensive cellular and animal experiments, aiming to provide new immunotherapeutic targets and improve prognosis.

Autophagy is closely linked to macrophage activation and polarization: it controls mitochondrial quality control and the metabolic reprogramming that accompanies M1-like pro-inflammatory activation, whereas impaired autophagy generally favors M2-like phenotypes (16, 17). This provides the biological rationale for evaluating whether TUBA1A-driven M1 polarization depends on autophagic activity.

## 2 Methods

### 2.1 Data acquisition

BUC gene expression profiles were from GEO (www.ncbi.nlm.nih.gov/geo) and TCGA (portal.gdc.cancer.gov). Dataset GSE13507 includes 67 adjacent normal and 165 primary bladder cancer tissues (no recurrent samples). GSE3167 has 14 normal and 46 primary bladder cancer tissues. TCGA contains 412 cases with cancer and paracancerous data. Single-cell transcriptome data were sourced from 11 bladder cancer fastq files labelled SRR12603780 to SRR12603790 in the BioProject database, including three single-cell samples of normal bladder tissue and eight single-cell samples of *in situ* bladder cancer.

### 2.2 Data preparation

BUC gene expression data were processed using R (4.2.2) and Bioconductor packages. Transcriptome files underwent probe ID conversion, standardization, and conversion to FPKM. Cell Ranger (7.0.0) generated a UMI matrix, converted to a Seurat (4.3.0) object. Cells with UMI <1000 or >20% mitochondrial/red blood cell UMIs were filtered. Post-QC, UMI counts were log-normalized (LogNormalize, scale factor = 10,000). Top 2000 variable genes were analyzed. PCA (top 20 PCs) reduced dimensions; Seurat’s FindClusters (res=2.5) identified 34 clusters (tSNE/UMAP visualization). Cells were grouped into 8 types via markers, then subclustered. FindAllMarkers identified differential genes. Clusters included mast (TPSAB1+), T (CD3D+), myeloid (LYZ+), epithelial (EPCAM+), fibroblast (LUM+), endothelial (VWF+), B (CD79A+), and smooth muscle (ACTA2+) cells.

### 2.3 NMF of aggrephagy-related genes in immune cells

To study the effect of aggrephagy on TME cells, we analyzed TME expression (44 aggrephagy-related genes in primary TME cells; source: MSigDB REACTOME_AGGREPHAGY (v7.5.1)) via dimensionality reduction, using scRNA matrix with screening, clustering, and NMF for subtypes; post-isolating clusters, FindAllMarkers (logFC 0.5) identified markers, feature genes (logFC >1, top aggrephagy-related genes as representatives) were selected, with DotPlot showing top genes per cluster and FeaturePlot illustrating the distribution of specific aggrephagy-related genes in the bladder cancer TME. NMF was performed with a fixed factorization rank of k = 10 (method “snmf/r”; seed “nndsvd”) on the expression matrix of the 44 aggrephagy-related genes.

### 2.4 Immune response analysis

We obtained gene expression data from TCGA and GEO databases. After removing normal samples, these data were analyzed with the Tumor Immune Dysfunction and Exclusion (TIDE) algorithm (18) to predict the response of bladder cancer patients to immunotherapy. Using the output data, we further explored the relationships between aggrephagy-related NMF signatures. Additionally, immune checkpoints collected from public datasets were tested against their corresponding datasets.

### 2.5 Cellchat analysis

The “cellchat” package (CellChatDB.human; Secreted Signaling; min.cells = 10) was used to explore the cellular interactions among the subtype clusters (19, 20).

### 2.6 Immunoinfiltration analysis

We performed immune infiltration analysis using the BEST online tool and the R package IOBR, including the algorithms CIBERSORT, ESTIMATE, quanTIseq, and xCell with default parameters, and conducted immune infiltration-related analysis of the TUBA1A gene in bladder cancer transcriptome data (21).

### 2.7 Metabolic analysis

We used the scMetabolism package (method “VISION”; KEGG pathway database) to assess the metabolic activity of each macrophage subset and plotted it as a bubble chart.

### 2.8 KEGG enrichment analysis

Using marker genes from aggrephagy-related clusters of different TME cell types, pathway enrichment analysis was performed with R packages (clusterProfiler and org.Hs.eg.db) against the KEGG database. Cell landscape enrichment maps visualized and clustered pathways; only gene sets with adjusted p-value < 0.05 were considered significant. Top three pathways of these clusters were focused on, followed by KEGG analysis of the TUBA1A gene.

### 2.9 M1/M2 macrophage polarization score

We used the “scMetabolism” package and the AddModuleScore function of the “Seurat” package (22) to score the macrophage subtypes using the M1/M2 macrophage-related gene set (source: MSigDB C7, CLASSICAL_M1_VS_ALTERNATIVE_M2_MACROPHAGE_UP/DN, with nbin = 12), and the scoring results were displayed using violin plots and UMAP feature plots.

### 2.10 Cell culture

This research used four bladder cancer cell lines (T24, HT-1376, RT-112, 5637) and one normal human bladder epithelial cell line (HCV-29), all from Shanghai’s Chinese Academy of Sciences Cell Bank. Cells were cultured in RPMI-1640 medium (Gibco, USA) with 10% FBS (Gibco, USA) in a 37 °C, 5% CO_2_ humidified incubator.

### 2.11 Cell transfection

T24 cells at 60% confluence were transduced with the TUBA1A overexpression lentivirus(from Shanghai GeneChem Co., Ltd.) or the empty vector lentivirus in the presence of polybrene (5 μg/ml) at an MOI of 5. Stable pools were selected with 1.0 μg/ml puromycin for 14 days, and the same selection was applied to cells transduced with the empty vector. Overexpression was verified by qRT-PCR and western blotting. Cells transduced with the TUBA1A overexpression lentivirus and with the empty vector lentivirus are hereafter referred to as TUBA1A-OE and NC-OE, respectively.

### 2.12 qRT-PCR

Total RNA from logarithmic phase cells (5.0×10^5–1.0×10^6) was extracted using RNAeasy™ Kit (Beyotime) with modified protocol; purity (A260/A280 1.8–2.1) and stored at -80 °C. First-strand cDNA was synthesized from 1 μg RNA using NovoScript^®^ Plus Kit (Novoprotein) and stored at -20 °C. qRT-PCR used NovoStart^®^ SYBR SuperMix (Novoprotein) in 20 μL reactions (2×SYBR, 1.0 μM primers, 1–2 μL cDNA) with cycling: 95 °C for 1 min, then 45 cycles of 95 °C/20s and 55 °C/30s. TUBA1A and GAPDH (reference) primers were used; relative expression was calculated via 2^(-ΔΔCt) with 3 repetitions.

Sequence (5’→3’),
CD86-F GAGTGAACAGACCAAGAAAAGAGAA
CD86-R AAAAACACGCTGGGCTTCATC
CD206-F AGCCAACACCAGCTCCTCAA
CD206-R CAAAACGCTCGCGCATTGTC
IL-12-F ACCCTGACCATCCAAGTCAAA
IL-12-R TTGGCCTCGCATCTTAGAAAG
ARG1-F TCATCTGGGTGGATGCTCAC
ARG1-R GAGAATCCTGGCACATCGG
TUBA1A-F GTTGATTATGGCAAGAAGTCC
TUBA1A-R TCAGAGTGCTCCAGGGTG
GAPDH-F ATCATCAGCAATGCCTCC
GAPDH-R CATCACGCCACAGTTTCC

### 2.13 CCK-8 assay

Cells were plated into 96-well plates (Corning, Corning, NY, USA) at a seeding density of 3 × 10³ cells per well and placed in a humidified incubator. To prevent medium evaporation, PBS was added to the surrounding areas of the plate. At predetermined time intervals, 100 µL of culture medium containing 10% CCK-8 (EnoGeneCell, Nanjing, China) was added to each well. Following a 2-hour incubation at 37 °C, the optical density (OD) was measured over three consecutive days. Each sample underwent three replicate tests.

### 2.14 Western blotting

Cells were harvested and lysed in RIPA buffer (Beyotime, Shanghai) with protease inhibitor PMSF (Beyotime, Shanghai) for enhanced efficiency. Protein concentration was measured via BCA kit (Beyotime, Shanghai). Proteins (30μg/sample) were separated by 10% SDS-PAGE, transferred to a membrane, and blocked immediately for 15min. Primary antibodies (GAPDH 1:25000, 60004-1-lg; TUBA1A 1:50000, 67840-1-lg; 19532-1-AP) were incubated overnight at 4 °C on a shaker. After secondary antibody incubation (2h, RT), membranes were washed with TBST, developed with ECL reagent (NCM Biotech, Suzhou), and imaged. Band intensities were quantified using ImageJ. Western blot signals were acquired within the linear detection range to ensure reliable quantification.

### 2.15 EdU cell proliferation assay

The Cell-Light™ EdU kit (RiboBio, China) was used to evaluate cell proliferation, following the guidelines provided by the manufacturer. In brief, cells were exposed to EdU reagent for 2 h, followed by fixation with paraformaldehyde for 30 min, and neutralization of excess aldehydes with glycine for 5 min. The cells were then stained with Apollo 567 and Hoechst 33342, each for a duration of 30 min. A fluorescence microscope was used to capture images of the cells, and the proliferation rate was quantified using ImageJ software.

### 2.16 Transwell invasion assay

Cell invasion assay: 24-well transwell chambers (Corning) with Matrigel. Serum-starved (12h) logarithmic-phase TUBA1A-OE/NC-OE cells (trypsinized, centrifuged, resuspended) – 6×10^4^ (250μL) added to upper chambers; lower chambers with 750μL 30% FBS medium. Incubated 48-72h (37 °C/5% CO_2_), lower cells fixed (4% paraformaldehyde, 15min RT) and stained (1% crystal violet, 15min RT). Post-rinse, residual upper cells removed; inserts dried. Invaded cells counted (5 fields/chamber, ImageJ) in triplicate. Images (200×) and graphs generated.

### 2.17 Wound healing assay

T24 cells were cultured in 6-well plates until reaching 80% confluence. Scratches were manually created by disrupting a small area of the cell monolayer with a pipette tip. Images were taken immediately after the initial scratch and again 24 hours later to monitor cell migration and wound healing. The images were analyzed to determine the cell migration rate.

### 2.18 Cell apoptosis assay

Logarithmic-phase TUBA1A-OE and NC-OE cells were used. Medium was transferred; cells were PBS-washed, trypsinized (0.25% EDTA-free) until edge retraction, digestion stopped, medium added to dislodge cells. The suspension was centrifuged (1000×g, 5 min), the supernatant discarded, and the cells PBS-resuspended and counted. 5×10^4^-1×10^5^ cells were centrifuged (1000×g, 5 min); 195 μl binding buffer was added, followed by 5 μl Annexin V-APC and 10 μl PI, and the cells were incubated for 15 min at 20-25 °C in the dark. Double fluorescence was detected by flow cytometry, and the apoptosis rate was calculated from the quadrant populations (early plus late apoptotic cells).

### 2.19 Autophagy electron microscope

20 μl TUBA1A-OE cell suspension was dropped on a carbon film copper grid, stood for 3–5 min, residual liquid absorbed with filter paper; 2% phosphotungstic acid (pH 6.8) was added to cover the grid for 1–2 min, stained solution absorbed similarly, dried at RT for 30 min; observed under JEM-1400Flash transmission electron microscope (80 kV), 5 random fields imaged to analyze autophagosome number and morphology.

### 2.20 Macrophage polarization induction

Human monocytic leukemia THP-1 cells were induced to M0 macrophages with PMA (100 ng/ml, 12h), then to M1 (LPS) and M2 (IL-4) macrophages, grouped as M0, M1, M2. Detected: M1 markers CD86/IL-12 (flow cytometry, WB); M2 markers CD206/Arg1 (flow cytometry, WB); TUBA1A expression (PCR, WB) in three groups. Co-cultured M1 macrophages with T24 cells (groups: THP-1+PMA; THP-1+PMA+LPS (100ng/mL, 48h); THP-1+PMA+NC-OE; THP-1+PMA+TUBA1A-OE). Detected: bladder cancer cell proliferation (CCK-8), macrophage migration (Transwell), autophagy-related proteins P62/ATG5/LC3B (WB).

### 2.21 Preparation of conditioned medium and chloroquine treatment

Conditioned medium (CM) was collected from NC-OE and TUBA1A-OE cells. Briefly, when the cells reached approximately 70% confluence, the culture medium was replaced with serum-free RPMI-1640; after 24 h, the supernatant was collected, centrifuged (1,000 × g, 5 min) and filtered through a 0.22 μm filter. THP-1-derived M0 macrophages were then cultured with the indicated CM for 48 h. For autophagy inhibition, chloroquine (CQ, 20 μM) was added 1 h before CM stimulation and maintained throughout the incubation. Serum was added back to the collected CM before application to macrophages, so that the macrophages were not serum-starved during CM exposure. Four groups were used: M0 macrophages incubated with NC-OE CM (M0), with TUBA1A-OE CM (TUBA1A-OE M0), with NC-OE CM plus CQ (M0+CQ), and with TUBA1A-OE CM plus CQ (TUBA1A-OE M0+CQ).

### 2.22 Flow cytometry analysis of macrophage polarization markers

Macrophages in the indicated groups were harvested and washed twice with PBS. For surface staining, cells were incubated with fluorochrome-conjugated anti-CD86 and anti-CD206 antibodies or the corresponding isotype controls at 4 °C for 30 min in the dark. For intracellular staining of IL-12 and Arg1, cells were fixed, permeabilized and incubated with the corresponding antibodies according to the manufacturer’s instructions. Data were acquired on a flow cytometer and analyzed with FlowJo software. The percentages of marker-positive cells were recorded and used for statistical analysis.

### 2.23 Nude mouse tumorigenicity assay

All animal procedures were approved by the Institutional Animal Care and Use Committee of Jinzhou Medical University (Approval No. JYTFUDF201759) and were conducted in accordance with the ARRIVE guidelines. Male BALB/c nude mice (4–6 weeks old, 18–22 g) housed under specific pathogen-free conditions. TUBA1A-OE cells were used for subcutaneous tumor formation in nude mice, with the experiment divided into 2 groups (5 mice per group): the NC-OE group and the TUBA1A-OE group. The detections included: observing and recording the tumor growth curve and body weight; detecting the pathology of tumor tissues by H&E staining; detecting the expression level of TUBA1A in tumor tissues by PCR and WB; detecting TUBA1A and Ki67 by immunohistochemistry; and detecting the expression of autophagy-related proteins P62, ATG5 and LC3B by WB. Mice were randomly allocated to groups. Investigators were blinded to group allocation during tumor measurement and data analysis.

### 2.24 Data processing

Bioinformatics data were processed via R (4.3.0), and statistical analysis was done with GraphPad Prism 9.0. All experiments had 3 independent biological replicates. Data normality was tested by Shapiro–Wilk test. Two groups were compared by t-test; multiple groups by one-way ANOVA with *post-hoc* test; time-course data by two-way ANOVA. Bonferroni correction was used for multiple comparisons. Statistical significance was marked with asterisks (ns *P* > 0.05, *P < 0.05, **P < 0.01, ***P < 0.001), with *P*-values labeled in charts.

## 3 Result

### 3.1 Aggrephagy genes exhibit a strong immune response in the NMF subtype of macrophages

In the study, single-cell transcriptome data were initially partitioned into 8 clusters: Fibroblasts (LUM), T cells (CD3D), B cells (CD79A), Mast cells (TPSAB1), Smooth muscle cells (MYLK, ACTA2), Endothelial cells (PECAM1, VWF), Epithelial cells (EPCAM), and Myeloid cells (LYZ). This partitioning established a foundation for preliminary exploration of aggrephagy genes’ impact on the single-cell immune microenvironment (**Figure 1B**). Following application of the NMF algorithm, four cell clusters significantly influenced by aggrephagy genes were identified: Fibroblasts, CD8+T cells, B cells, and Macrophages. Subsequent gene subtype classification of each cluster yielded 19 gene signature-defined cell subtypes (**Figure 1C**).

**Figure 1:**
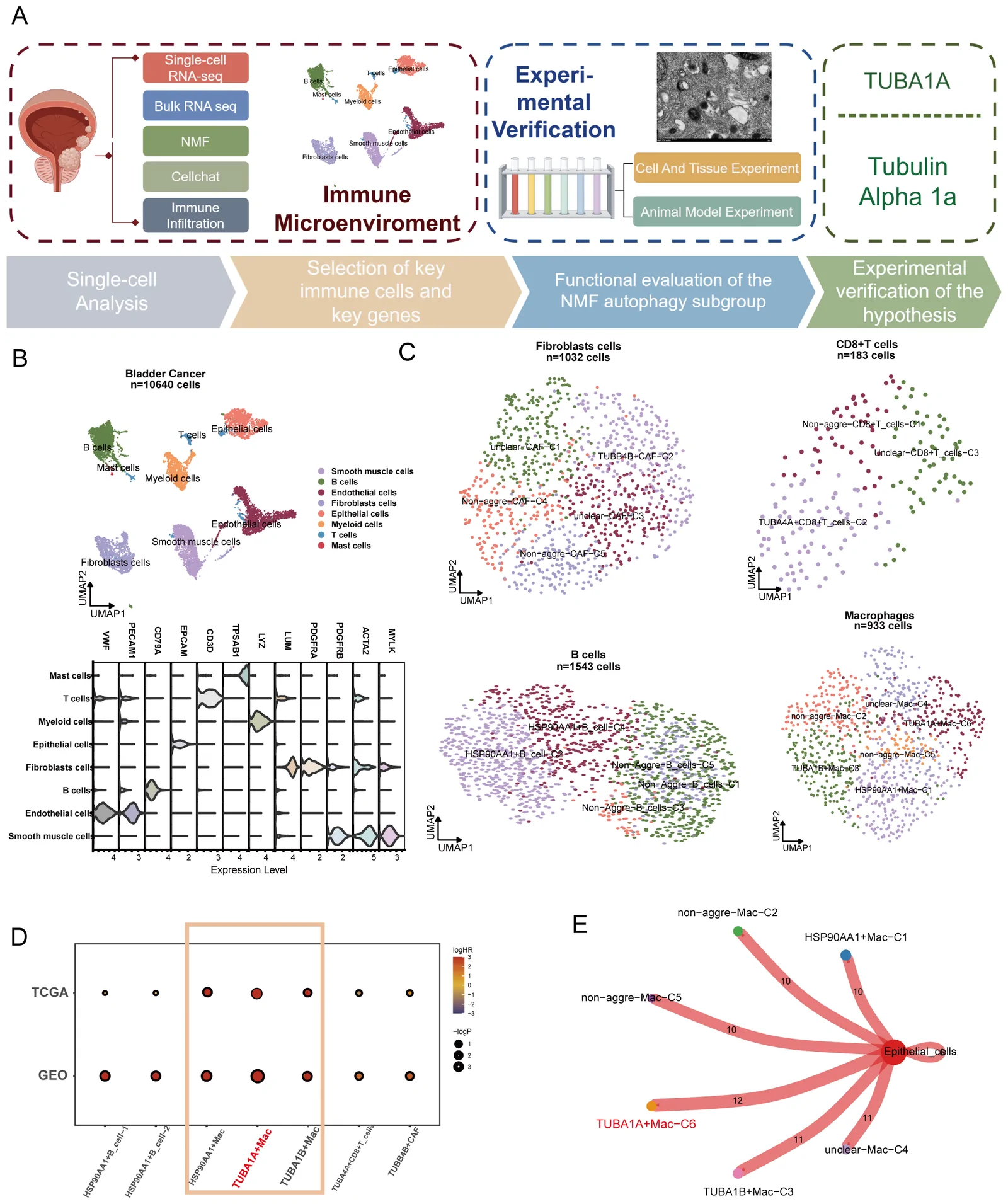
Aggrephagy-related genes tend to function within macrophages of bladder cancer. **(A)** Flowchart of the entire study, illustrating the overall workflow from single-cell transcriptomic analysis and NMF-based aggrephagy subtyping to immune infiltration, functional enrichment, and *in vitro*/*in vivo* experimental validation (created with Figdraw). **(B)** UMAP plot of single-cell transcriptome data showing 8 major cell subpopulations in bladder cancer (total 10,640 cells); each subpopulation was annotated by its canonical marker genes (Fibroblasts, LUM; T cells, CD3D; B cells, CD79A; Mast cells, TPSAB1; Smooth muscle cells, MYLK and ACTA2; Endothelial cells, PECAM1 and VWF; Epithelial cells, EPCAM; Myeloid cells, LYZ), and the violin plots below display the expression levels of the marker genes in each subpopulation. **(C)** UMAP plots of NMF clustering results based on 44 aggrephagy-related genes, generated separately for fibroblasts, CD8+ T cells, B cells, and macrophages, yielding 19 gene signature-defined cell subtypes. **(D)** Immune response analysis of the aggrephagy-related subtypes using the TIDE algorithm in the TCGA-BLCA and GSE13507 datasets, showing that aggrephagy-related gene signatures elicit the strongest immune response in macrophage subtypes, with the TUBA1A+Mac subtype being the most prominent. **(E)** CellChat analysis of intercellular communication between macrophage subtypes and bladder cancer tumor epithelial cells, showing that the TUBA1A+Mac-C6 cluster exhibits the strongest cell-communication activity.

After selecting aggrephagy-related gene clusters from these 19 subtypes, their immune responses were tested using TCGA data and GSE13507 data, respectively. Results revealed that aggrephagy genes exhibited the strongest signature immune response in macrophage subtypes, with the TUBA1A+Mac subtype being the most prominent (**Figure 1D**). CellChat analysis between all macrophage subtypes and bladder cancer tumor epithelium demonstrated that cell communication responses were most intense in the TUBA1A+Mac group (**Figure 1E**). Consequently, subsequent studies focused on investigating the mechanisms by which the TUBA1A gene regulates macrophages to influence bladder cancer occurrence and development.

### 3.2 The TUBA1A gene shows a strong positive correlation with M1 macrophages

First, fine classification of the macrophage cluster defined M1 macrophages, M2 macrophages, and remaining cells collectively referred to as monocytes. Among these, in bladder cancer, the proportion of M2 macrophages with low TUBA1A expression was increased compared to that in normal tissues (**Figure 2A**). Immune infiltration methods including CIBERSORT, ESTIMATE, Quantiseq, and xCell were then used to evaluate the TUBA1A gene, revealing that positive expression of TUBA1A had a relatively strong positive correlation with M1 macrophage activation, while exerting a weak effect on M2 macrophages (**Figure 2B**). In correlation analysis of TUBA1A with immunomodulatory factors, pro-inflammatory factors positively correlated with antigen presentation included HLA-B, HLA-DQA1, HLA-DRA, HLA-DPA1, HLA-C, HLA-DOA, HLA-DMB, HLA-DPB1, HLA-A, HLA-DRB1, etc.; positively correlated chemokines included CCL2, CCL3, CXCR3, CXCR5, CXCR1, CXCL9, CXCL10, etc.; and positively correlated immunostimulatory-related factors also included CD86 and IL2RA. These immunomodulatory factors were all closely related to M1 macrophage activation (**Figure 2C**). Meanwhile, metabolic enrichment analysis of the TUBA1A+Mac subtype showed that among all subtypes, TUBA1A+Mac exhibited a unique and strong metabolic profile, with high enrichment in pathways such as ketone body metabolism (the synthesis and degradation pathway of ketone bodies showed bright red and large dots; ketone body metabolism is related to macrophage plasticity and may support pro-inflammatory or tissue repair functions), steroid hormone biology (steroid hormone biosynthesis and steroid biosynthesis pathways were strongly enriched; steroids regulate immune responses, suggesting TUBA1A+Mac may be involved in endocrine-immune crosstalk), carbohydrate metabolism (multiple glucose/nutrient pathways including starch and sucrose metabolism, glycolysis/gluconeogenesis, and the pentose phosphate pathway were activated, indicating reliance on active glucose flux consistent with the high energy demand of specialized macrophage functions), and lipid and amino acid metabolism (sphingolipid metabolism, linoleic acid metabolism, and amino sugar and nucleotide sugar metabolism pathways were enriched, pointing to remodeling of membrane components and signaling molecules crucial for macrophage activation states) (**Figure 2D**).

**Figure 2:**
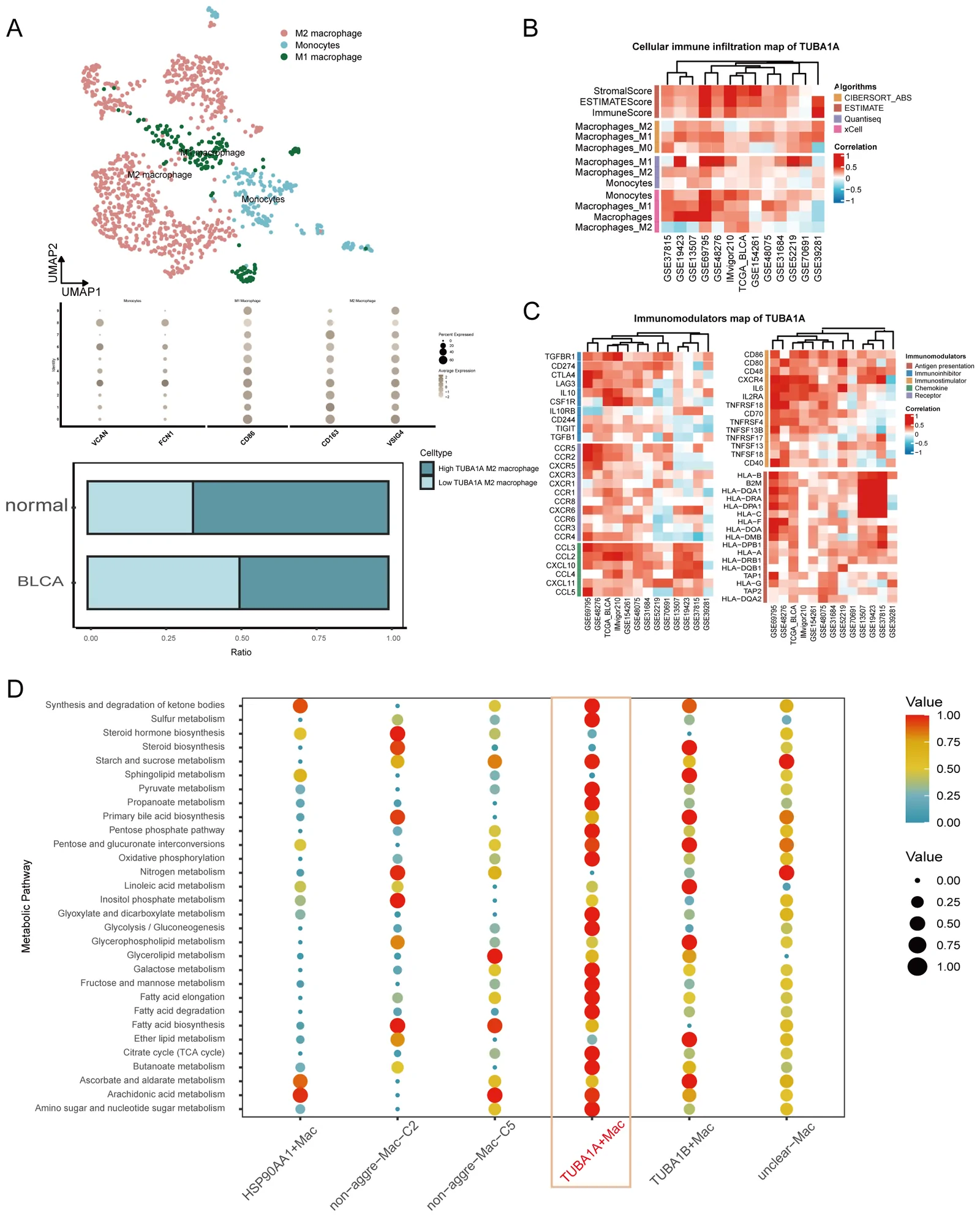
Association of TUBA1A with macrophage subsets, immune infiltration, immunomodulators, and metabolic pathways in bladder cancer. **(A)** UMAP plot showing the re-clustering of macrophages into M1 macrophages, M2 macrophages, and monocytes; the attached bar graph shows that M2 macrophages with low TUBA1A expression account for a relatively high proportion in bladder cancer tissues compared with normal tissues. **(B)** Immune infiltration analysis of TUBA1A using multiple algorithms (CIBERSORT, ESTIMATE, quanTIseq, and xCell, implemented with the BEST online tool and the IOBR R package), showing that TUBA1A expression is positively correlated with M1 macrophage activation; the correlation heatmap displays the relationship between TUBA1A and immune cell subsets (M1 macrophages, M2 macrophages, monocytes) across multiple datasets (GSE37815, GSE19423, GSE13507, GSE69795, GSE48276, IMvigor210, TCGA-BLCA, GSE154261, GSE48075, GSE31684, GSE52219, GSE70691, GSE39281). **(C)** Immunomodulator map showing the association between TUBA1A and immunomodulatory factors involved in M1 macrophage activation, including immunostimulators, immunoinhibitors, antigen presentation molecules (e.g., HLA-B, B2M, HLA-DQA1), chemokines and their receptors (e.g., CCR5, CXCR5, CCL2, CXCL10); the heatmap compares the expression differences between high- and low-TUBA1A groups in M2 macrophages, as well as between normal and bladder cancer samples, across datasets (GSE37815, GSE39281, GSE69795, GSE48276, TCGA-BLCA, IMvigor210). **(D)** Metabolic pathway map of the NMF subgroups assessed with the scMetabolism package (VISION method, KEGG pathway database), showing the activation levels (range 0.00-1.00) of multiple metabolic pathways, including oxidative phosphorylation, glycolysis/gluconeogenesis, citrate cycle (TCA cycle), fatty acid metabolism, steroid biosynthesis, and ketone body synthesis and degradation, in the TUBA1A+Mac subgroup.

### 3.3 Enrichment analysis revealed that the TUBA1A gene was associated with the immune regulation of macrophages

Expression analysis confirmed low expression of the TUBA1A gene in bladder cancer tumors (**Figure 3A**). Cell functions in the TUBA1A+Mac cluster are enriched in Oocyte meiosis, Gap junction, and Cell cycle (**Figure 3B**). KEGG enrichment analysis of TUBA1A and related genes revealed enrichment in the Chemokine signaling pathway, which is positively correlated with M1 macrophage polarization. Additionally, the Fc epsilon RI signaling pathway assists in M1 macrophage polarization, while Purine metabolism and Pyrimidine metabolism provide nucleic acids and energy raw materials to meet the high metabolic demands of M1 macrophage polarization. Beyond regulating M1 macrophage polarization, specific disease enrichment also yielded results related to Bladder cancer. Meanwhile, the Cell cycle, Notch signaling pathway, and ErbB signaling pathway regulate tumor proliferation/cycle progression in bladder cancer. Pathways such as DNA replication and Non-homologous end-joining are involved in the accumulation of gene mutations and can drive bladder cancer occurrence. Pathways including Fatty acid metabolism and Glycolysis/Gluconeogenesis can supply energy for bladder cancer via the Warburg effect. All these lines of evidence indicate that high TUBA1A expression can activate M1 macrophages, thereby inhibiting cancer occurrence. It is thus inferred that low TUBA1A expression in bladder cancer is highly likely to reduce M1 macrophage polarization, while relatively increasing macrophage M2 polarization responses in the bladder cancer immune microenvironment, thereby contributing to bladder cancer occurrence and development (**Figure 3C**). Subsequently, a series of experiments verified the connection between the bladder cancer phenotype identified in enrichment analysis and the TUBA1A gene. Background expression testing confirmed that TUBA1A is indeed lowly expressed in bladder cancer (**Figure 3D**). The T24 cell line, exhibiting the optimal effect, was used to construct the TUBA1A-OE cell line, and WB was employed to detect protein expression levels following overexpression (**Figure 3E**). The CCK-8 experiment demonstrated that TUBA1A overexpression inhibited cell proliferation (**Figure 4C**).

**Figure 3:**
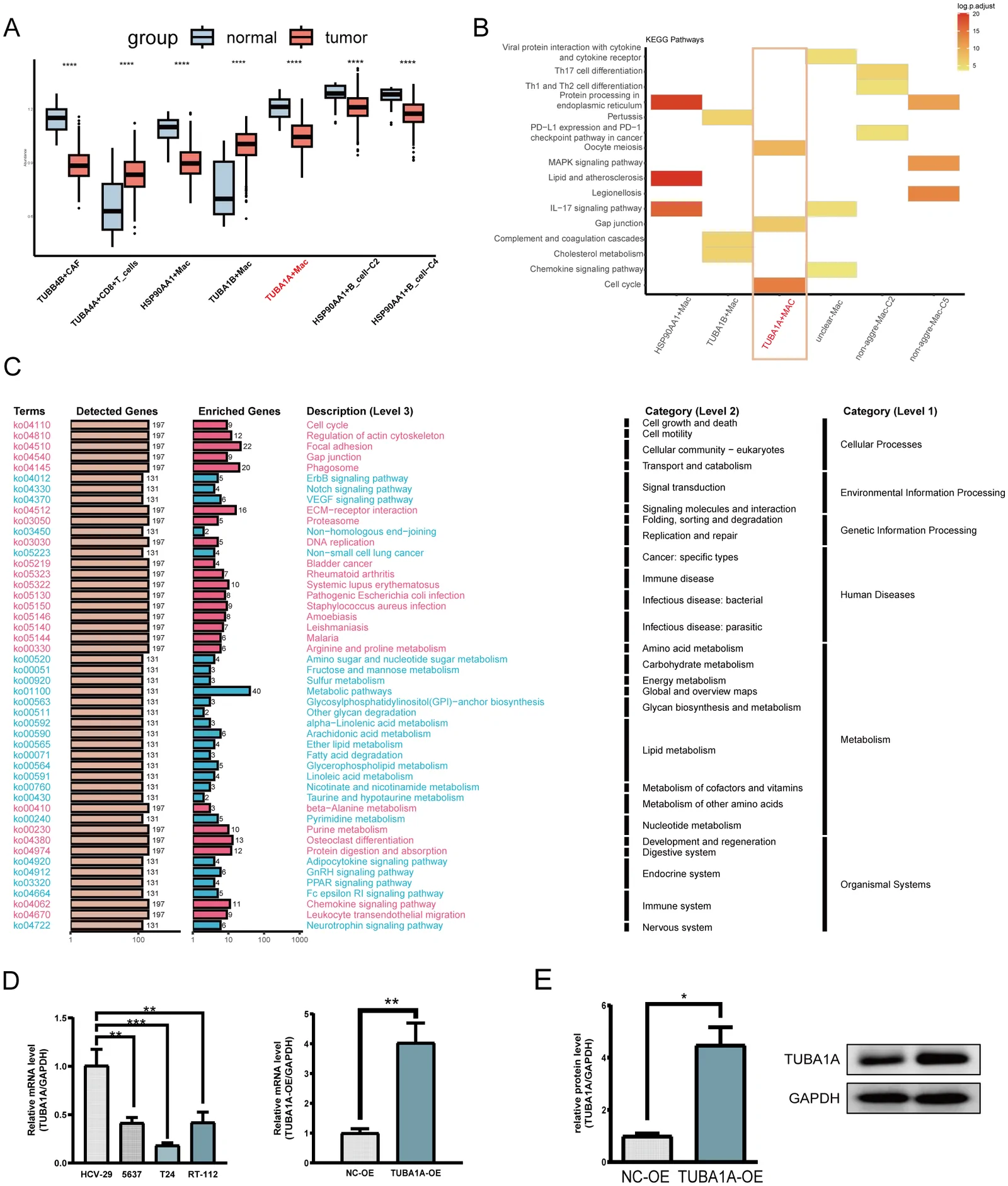
Expression, functional enrichment, and experimental validation of TUBA1A in bladder cancer. **(A)** Box plot showing the results of the normal-tumor (NT) differential expression analysis, indicating that TUBA1A is lowly expressed in tumor tissues compared with normal tissues; each dot represents one sample. **(B)** Functional enrichment analysis (clusterProfiler R package, adjusted P < 0.05) of the TUBA1A+Mac cluster, displaying significantly enriched KEGG pathways, including “Oocyte meiosis”, “Gap junction”, and “Cell cycle”. **(C)** KEGG pathway enrichment results presented as a three-level hierarchical structure centered on TUBA1A-related functions: level 1 categories (e.g., Cellular Processes, Environmental Information Processing, Metabolism, Human Diseases); level 2 subcategories (e.g., “Cell growth and death”, “Cancers: specific types”, “Immune disease”); and level 3 specific terms (e.g., “Cell cycle”, “Focal adhesion”, “Bladder cancer”), together with the number of detected genes in each term. **(D)** qRT-PCR results showing the baseline mRNA expression of TUBA1A (normalized to GAPDH) in bladder cancer cell lines (HCV-29, 5637, T24, RT-112) and the relative mRNA level after TUBA1A overexpression (TUBA1A-OE) compared with the negative control (NC-OE); data are from three independent biological replicates and are presented as mean ± SD (Student’s t-test). **(E)** Western blot analysis verifying the protein expression of TUBA1A in the NC-OE and TUBA1A-OE groups, with GAPDH as the internal loading control (*P < 0.05, **P < 0.01, ***P < 0.001, ****P < 0.0001).

**Figure 4:**
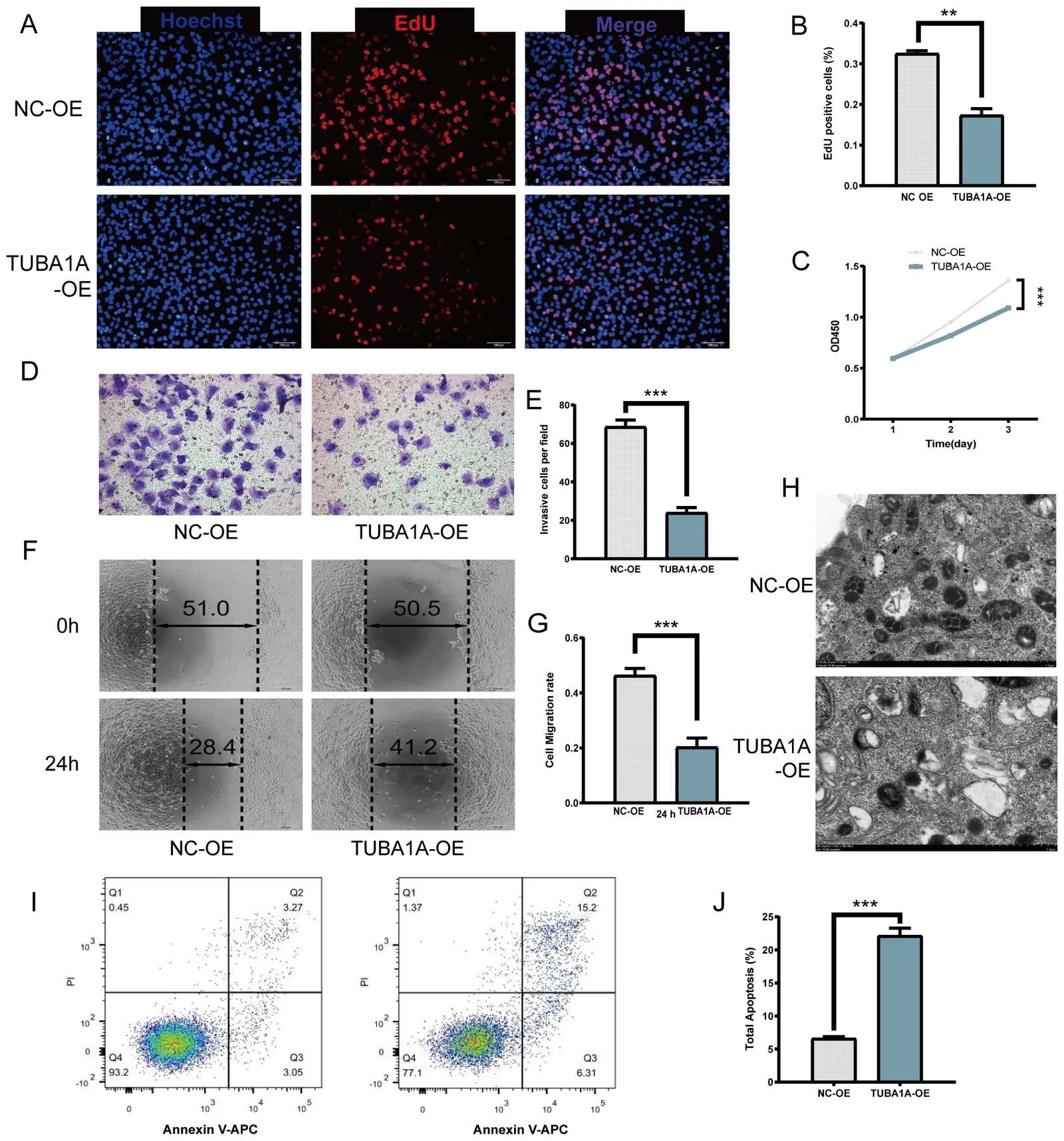
Effects of TUBA1A overexpression on cell proliferation, migration, invasion, and apoptosis **(A)** Representative images of the EdU assay showing EdU-positive (proliferating) cells in NC-OE and TUBA1A-OE groups, with Hoechst nuclear counterstaining and merged images **(B)** Statistical graph of the EdU assay, showing the percentage of EdU-positive cells in NC-OE and TUBA1A-OE groups **(C)** CCK-8 assay results showing the cell proliferation activity (OD450) of NC-OE and TUBA1A-OE groups over time (days) **(D)** Representative images of the Transwell invasion assay in NC-OE and TUBA1A-OE groups **(E)** Statistical graph of the Transwell invasion assay, showing the number of invasive cells per field in NC-OE and TUBA1A-OE groups **(F)** Representative images of the wound-healing assay showing cell migration in NC-OE and TUBA1A-OE groups **(G)** Statistical graph of the wound-healing assay, presenting the cell migration rate in NC-OE and TUBA1A-OE groups **(H)** Transmission electron microscopy images of autophagy in NC-OE and TUBA1A-OE groups, visualizing autophagic structures (e.g., autophagosomes) **(I)** Flow cytometry results of the apoptosis assay using Annexin V staining, showing cell population distributions (Q1: necrotic; Q2: late apoptotic; Q3: live; Q4: early apoptotic) in NC-OE and TUBA1A-OE groups **(J)** Statistical graph of the apoptosis assay, quantifying the total apoptosis rate in NC-OE and TUBA1A-OE groups (**P < 0.01, ***P < 0.001).

### 3.4 Overexpression of TUBA1A can affect multiple phenotypes of bladder cancer

Using the TUBA1A-OE stably transfected cell line, specific phenotypic experiments were conducted based on the results of enrichment analysis. The EdU experiment results showed that TUBA1A overexpression inhibited the proliferation of bladder cancer cells (**Figures 4A, B**). The CCK-8 assay showed that TUBA1A overexpression inhibited cell proliferation over time (**Figure 4C**). The Transwell experiment indicated that the invasive ability of bladder cancer cells decreased following TUBA1A overexpression (**Figures 4D, E**). The wound healing assay demonstrated that the migration ability of bladder cancer cells was reduced after TUBA1A overexpression (**Figures 4F, G**). Autophagy electron microscopy experiments revealed that after TUBA1A overexpression, the volume and number of autophagic vacuoles in the electron microscope field of view increased significantly, indicating an enhancing effect on autophagic activity (**Figure 4H**). Cell apoptosis experiments showed that the apoptotic ability of cells increased significantly following TUBA1A overexpression (**Figures 4I, J**).

### 3.5 Cell induction experiments indicated that high expression of TUBA1A promoted the production of M1 macrophages

Scoring analysis of M1/M2 macrophages showed that the M1 macrophage activation score was relatively high in the TUBA1A+ Mac group, whereas the M2 macrophage activation score was relatively low (**Figure 5A**). UMAP feature plots further showed a relatively high activation degree of M1 macrophages in the region associated with TUBA1A+Mac (**Figure 5B**). To verify the activating effect of the TUBA1A gene on M1 macrophages, a macrophage polarization experiment was designed (**Figure 5C**).

**Figure 5:**
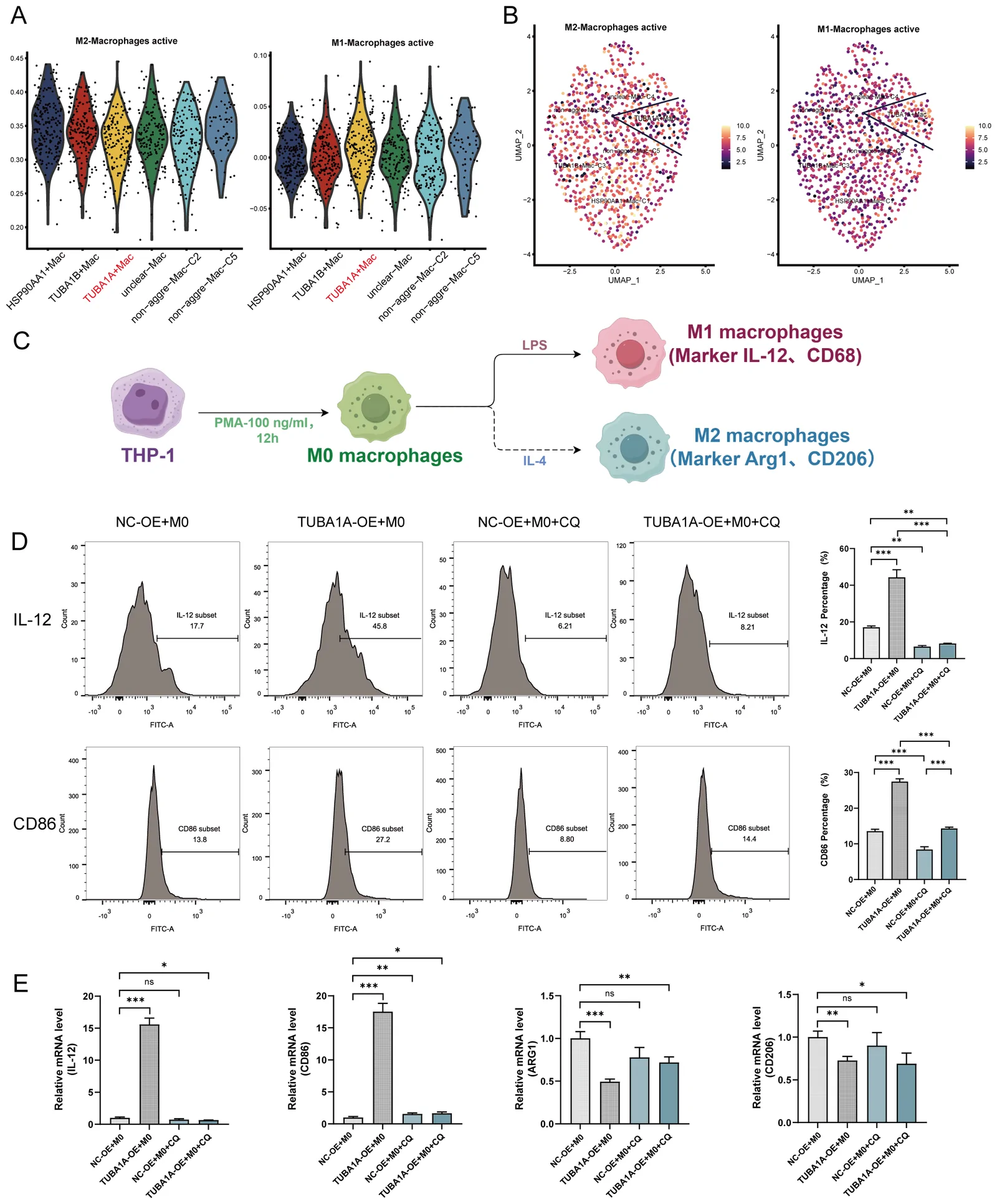
Association of the TUBA1A-positive macrophage subgroup with macrophage polarization and effects of TUBA1A-OE conditioned medium on macrophage polarization and autophagy **(A)** Violin plots showing the activity scores of M1- and M2-like macrophages across the indicated macrophage subgroups, with the TUBA1A+Mac subgroup highlighted. **(B)** UMAP feature plots showing the distribution of M1- and M2-macrophage activity scores across the macrophage subgroups. **(C)** Schematic illustration of the THP-1-derived macrophage induction procedure. THP-1 cells were treated with PMA to generate M0 macrophages, which were then induced toward an M1-like phenotype with LPS or an M2-like phenotype with IL-4. **(D)** Flow-cytometry results showing the percentages of IL-12- and CD86-positive macrophages after treatment with control conditioned medium, TUBA1A-OE conditioned medium, chloroquine (CQ), or TUBA1A-OE conditioned medium combined with CQ. **(E)** Relative mRNA expression of the M1 markers IL-12 and CD86 and the M2 markers ARG1 and CD206 in the indicated treatment groups, as determined by qRT-PCR and normalized to GAPDH. *P < 0.05, **P < 0.01, ***P < 0.001, ns *P* > 0.05.

Flow cytometry confirmed the success of cell induction: the proportions of cells positive for the M1 markers IL-12 and CD86 were significantly higher in the M1 group than in the M0 and M2 groups (**Supplementary Figure S1A**), whereas the proportions of cells positive for the M2 markers Arg1 and CD206 were significantly higher in the M2 group than in the other two groups (**Supplementary Figure S1C**). WB confirmed that the CD86 and IL-12 protein levels were higher in the M1 group (**Supplementary Figure S1B**) and that the CD206 and Arg1 protein levels were higher in the M2 group (**Supplementary Figure S1D**).

### 3.6 Autophagy inhibition abolishes the TUBA1A-induced M1-like polarization of macrophages

To determine whether TUBA1A secreted by bladder cancer cells directly regulates macrophage polarization and whether this effect depends on autophagic activity, NC-OE CM and TUBA1A-OE CM were applied to THP-1-derived M0 macrophages in the presence or absence of the autophagy inhibitor chloroquine (CQ). Flow cytometry showed that the percentages of IL-12- and CD86-positive cells were markedly increased after incubation with TUBA1A-OE CM compared with NC-OE CM, whereas the addition of CQ abolished this increase (**Figure 5D**). Consistent with the flow cytometric results, qRT-PCR showed that the mRNA levels of the M1 markers IL-12 and CD86 were markedly increased by TUBA1A-OE CM and were restored to control levels by CQ treatment. In contrast, TUBA1A-OE CM decreased the mRNA levels of the M2 markers ARG1 and CD206. The reduction in ARG1 was reversed by CQ, and the decrease in CD206 was not restored by CQ (**Figure 5E**). Collectively, these results suggest that TUBA1A-OE bladder cancer cells promote M1-like polarization of macrophages through a mechanism sensitive to autophagy inhibition.

### 3.7 TUBA1A has an inhibitory effect on bladder cancer

LPS and the TUBA1A-OE cell line were added to induced M0 macrophages, respectively. Subsequently, decreased cell proliferation ability was observed in both the group induced into M1 macrophages by LPS addition and the group with TUBA1A-OE cell line addition (**Figure 6A**). Similarly, in the Transwell experiment, fewer cells were noted in the M0 cell group with TUBA1A-OE addition compared to other groups, indicating that the TUBA1A gene exerts an anti-cell proliferation effect similar to LPS, potentially inducing M1 polarization in M0 macrophages (**Figure 6B**). Results of the Transwell experiment were visualized via column statistical charts (**Figure 6C**). WB was then used to detect the expression levels of autophagy-related proteins SQSTM1/p62, ATG5, and LC3B, revealing that in experimental groups, ATG5 and LC3B expression levels in PMA+LPS and PMA+ TUBA1A-OE groups were higher than those in the control group, while SQSTM1/p62 expression was lower. This confirms that TUBA1A functions similarly to LPS, exerting a positive effect on inducing M0 macrophage polarization toward M1 (**Figure 6D**). In nude mouse tumor formation experiments, tumor weight and size in the TUBA1A-OE group were significantly smaller than those in the control group (**Figure 6E**). PCR detection results supported the success of the nude mouse tumor formation experiment (**Figure 6F**). WB experiments on tumor tissues showed that in experimental groups, ATG5 and LC3B protein expression levels in tumor tissues were higher than those in the control group, while SQSTM1/p62 protein expression was lower, indicating that high TUBA1A expression can also positively regulate autophagy-related proteins in nude mice and promote autophagic activity. Quantification of autophagy-related proteins was visualized via column statistical charts (**Figure 6G**). Finally, tumor tissues were subjected to HE and immunohistochemical staining. HE staining results indicated that TUBA1A overexpression may inhibit tumor cell proliferation. Immunohistochemical staining confirmed successful TUBA1A overexpression, with dense brown staining and significantly increased protein levels in the TUBA1A-OE group. Additionally, reduced numbers of Ki67 cell proliferation markers in the TUBA1A overexpression group indicated that TUBA1A overexpression inhibited the proliferation ability of bladder cancer cells (**Figure 6H**).

**Figure 6:**
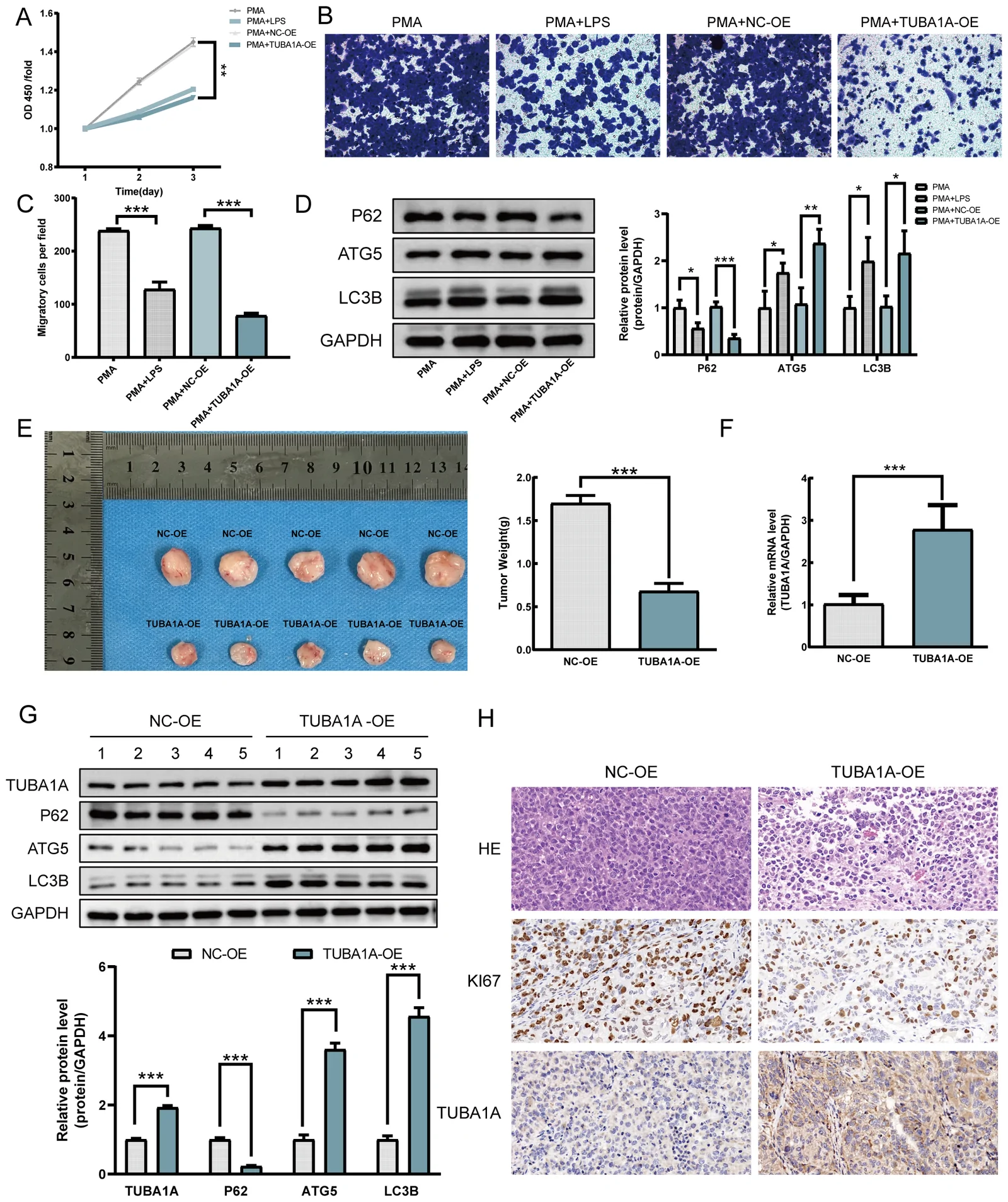
Effects of TUBA1A-OE bladder cancer cells on macrophage polarization and xenograft tumor growth *in vivo* **(A)** CCK-8 assay results showing cell viability (OD450/fold) in groups treated with PMA, PMA+LPS, PMA+NC-OE, and PMA+TUBA1A-OE over time **(B)** Transwell assay results displaying the number of migratory cells in PMA, PMA+LPS, PMA+NC-OE, and PMA+TUBA1A-OE groups **(C)** Bar graph quantifying the results of the Transwell assay, showing the number of migratory cells per field in PMA, PMA+LPS, PMA+NC-OE, and PMA+TUBA1A-OE groups **(D)** Western blot images and corresponding bar graphs of autophagy-related proteins (P62, ATG5, LC3B) in PMA, PMA+LPS, PMA+NC-OE, and PMA+TUBA1A-OE groups, with relative protein levels normalized to GAPDH **(E)** Images of tumor tissue blocks from nude mouse xenograft experiments and corresponding bar graphs showing tumor weights in NC-OE and TUBA1A-OE groups **(F)** Bar graph of PCR results quantifying TUBA1A overexpression levels in tumor tissue blocks from nude mouse experiments (relative mRNA level normalized to GAPDH) **(G)** Western blot images of autophagy-related proteins (TUBA1A, SQSTM1/p62, ATG5, LC3B) in tumor tissues from NC-OE and TUBA1A-OE groups, with corresponding bar graphs of relative protein levels (normalized to GAPDH) **(H)** Staining images of tumor tissue sections from nude mouse experiments, including HE staining and TUBA1A/Ki67 immunohistochemical staining (*P < 0.05, **P < 0.01, ***P < 0.001).

## 4 Discussion

BUC has a complex immune microenvironment, but its relationship to autophagy is still not fully understood. Although existing studies have revealed the role of immune cells in the progression of BUC, comprehensive research on integrating immune microenvironment factors and autophagy pathways is still limited (12). In this study, we identified a subset of macrophages with TUBA1A as the core through a combination of single-cell and holistic transcriptome analysis, revealing its key role in the BUC immune response. Single-cell approaches have increasingly been used to resolve immune and stromal cell states that shape immunotherapy response across tumor types (23). Non-negative matrix factorization of single-cell transcriptomes has similarly been used to resolve transcriptionally distinct subpopulations in other tumor types (24). TUBA1A encodes an α-tubulin isoform involved in cytoskeletal dynamics and autophagy-associated processes. Through CellChat analysis, we found that the TUBA1A+Mac subset had the strongest immune response and cellular communication activity. We also found that TUBA1A is significantly downregulated in tumor tissues, and its upregulation can significantly inhibit the malignant phenotype of BUC, including reducing proliferation, migration, and invasion, while promoting apoptosis (25, 26). These findings not only enhance our understanding of macrophage polarization in BUC, but also provide new insights into TUBA1A as a potential therapeutic target for immune microenvironment remodeling and tumor progression inhibition.

Unlike most previous studies that focus on correlation analysis, our work found that TUBA1A was positively correlated with the activation of M1 macrophages through multi-algorithm immunoinfiltration analysis, and the proportion of M2 macrophages in tumor samples with low TUBA1A expression was increased (27, 28). This study not only completed the correlation analysis, but also provided experimental evidence supporting a potential link between “immune polarization-autophagy-tumor phenotype” through polarization induction, co-culture, and functional verification of autophagy pathway proteins (SQSTM1/p62/ATG5/LC3B). This longitudinal chain of evidence goes beyond previous studies that rely solely on correlation, and suggests a key role of TUBA1A in the immune microenvironment, possibly by modulating macrophage polarization and autophagic activity (29–31). In addition, TUBA1A inhibits tumor progression through the remodeling of the immune microenvironment, further supporting its potential as an immunotherapy target (32, 33).

Immune infiltration and immunomodulator analysis showed that high expression of TUBA1A was associated with antigen presentation and up-regulation of inflammatory chemokines, and was positively correlated with the M1 polarization phenotype. Specifically, high expression of TUBA1A is closely related to the upregulation of HLA-like molecules, CD86, IL2RA, and the CCL/CXCL chemokine axis, which play an important role in M1 macrophages (34). Further polarization experiments showed that the expression of TUBA1A was significantly increased after M1 induction, while down-regulated after M2 induction (35, 36). These results support the important role of TUBA1A in immune polarization, suggesting that TUBA1A may improve tumor immune ecology by promoting the transformation of M0 macrophages to M1 polarization (36). In addition, TUBA1A also enhances the immune surveillance function of M1 macrophages by regulating the immunoreactivity of macrophages, promoting antigen presentation and the release of inflammatory factors (37).

At the molecular mechanism level, in bladder cancer cells TUBA1A upregulation was accompanied by the degradation of P62 and the increase of ATG5 and LC3-II, and the increase of autophagosomes was observed by transmission electron microscopy (TEM), further verifying the activation of TUBA1A on the autophagy pathway (33). Activation of autophagy pathway plays a key role in macrophage function, especially in the process of immune response and remodeling of tumor immune microenvironment (34). Metabolic analysis further revealed that TUBA1A+Mac cells exhibited significant metabolic signatures in pathways such as glycolysis, fat metabolism, and steroidal metabolism, which were highly consistent with the high energy demand state of M1 macrophages (35). These findings suggest that TUBA1A not only enhances M1 polarization through coupling with autophagy and metabolism, but also supports the anti-tumor effects of macrophages through metabolic reprogramming, thereby playing multiple roles in the tumor immune microenvironment (36). Of note, the relationship between autophagy and macrophage polarization is context-dependent: while several studies reported that autophagy supports M1-like pro-inflammatory activation (38), others showed that autophagy deficiency can also promote M1 polarization (17), indicating that the direction of this link may depend on the specific cell type and stimulus.

Whether a comparable autophagy-dependent program operates during macrophage–tumor cell crosstalk is a separate question. Macrophages in the tumor microenvironment are continuously exposed to factors released by neighboring malignant cells, and the conditioned-medium experiments argue that TUBA1A is one such factor. Macrophages cultured with medium conditioned by TUBA1A-OE bladder cancer cells acquired an M1-like phenotype, with IL-12 and CD86 induced at both mRNA and protein levels. When autophagic flux was inhibited in the responding macrophages, the induction of both markers was lost. Autophagic activity in the macrophage therefore appears to be required for TUBA1A-driven M1-like polarization, consistent with the proposed role of autophagy in the metabolic reprogramming and mitochondrial quality control that accompany M1-like activation (16, 38). Read together with the cell-intrinsic observations above, this suggests that TUBA1A acts at two levels — sustaining autophagic activity within the cell and, through factors released by tumor cells, favoring an M1-like state in the surrounding myeloid compartment. It should also be noted that the conditioned medium approach captures paracrine signaling from tumor cells but does not account for direct cell-cell contact between tumor cells and macrophages, which may additionally contribute to the polarization observed here.

*In vivo* experiments further consolidate these findings: in a nude mouse tumorigenesis model, TUBA1A overexpression significantly inhibited tumor growth, manifested by a significant reduction in tumor volume and weight, as well as a significant decrease in Ki67-labeled cell proliferation (39, 40). In addition, the overexpression of TUBA1A was consistent with changes in autophagy pathway proteins, with P62 degradation and ATG5 and LC3B increases, indicating that TUBA1A is involved in tumor suppression by activating the autophagy pathway (41). These results provide strong *in vivo* evidence for the role of TUBA1A in the tumor immune microenvironment.

At the same time, combined with the existing immune-related literature, we found that the high expression of TUBA1A may provide new ideas for immunotherapy. A dual mode of action, in which a single molecular axis acts directly on tumor cells while simultaneously reprogramming the tumor immune microenvironment, has been described in other tumor types and is regarded as an attractive therapeutic configuration (42). Its role in the tumor immune microenvironment, especially in promoting M1 macrophage polarization, regulating autophagy, and reshaping immune responses, suggests that TUBA1A may be a potential target in immunotherapy (43). TUBA1A not only modulates the tumor immune microenvironment, but may also serve as a key biomarker in combined immune strategies to improve immunotherapy response in bladder cancer patients (44, 45). Emerging evidence indicates that the spatial organization and metabolic states of the tumor immune microenvironment critically modulate immunotherapy benefit (46, 47).

Although this study reveals the potential role of TUBA1A in the immune microenvironment of bladder cancer, several of its limits follow from the funding and instrumentation available to us. TUBA1A expression was profiled across four bladder cancer cell lines and a normal urothelial line, while the functional and polarization experiments were carried out in T24 cells and THP-1-derived macrophages (48), and the xenograft experiments were performed in immunodeficient mice. More demanding models, such as primary tumor-associated macrophages and immunocompetent hosts, lay beyond the resources available to this study. In addition, THP-1-derived macrophages, although widely used and well characterized, do not fully recapitulate the phenotype and function of tumor-associated macrophages *in vivo*.

In summary, the present work places TUBA1A at the interface between aggrephagy and the immune microenvironment of bladder cancer. In tumor cells, loss of TUBA1A accompanies reduced autophagic activity, and restoring its expression constrains proliferation, migration and invasiveness *in vitro* as well as xenograft growth *in vivo*, while the same autophagic activity appears necessary for the M1-like polarization of macrophages exposed to tumor-derived factors. TUBA1A therefore merits evaluation as a candidate biomarker and as a target for approaches aimed at restoring macrophage-mediated control of bladder cancer.

## Data Availability

The original contributions presented in the study are included in the article/**Supplementary Material**. Further inquiries can be directed to the corresponding authors.

## Supplementary material

Supplementary Figure 1 Validation of M1- and M2-like macrophage induction in THP-1-derived macrophages **(A)** Flow-cytometry results showing the percentages of IL-12- and CD86-positive cells in the negative-control/M0, LPS-induced M1, and IL-4-induced M2 groups. **(B)** Representative western blot images and quantification of the M1 markers CD86 and IL-12 in M0, M1, and M2 macrophages, with GAPDH as the loading control. **(C)** Flow-cytometry results showing the percentages of ARG1- and CD206-positive cells in the negative-control/M0, LPS-induced M1, and IL-4-induced M2 groups. **(D)** Representative western blot images and quantification of the M2 markers CD206 and ARG1 in M0, M1, and M2 macrophages, with GAPDH as the loading control (****P* < 0.001).
