# Supplementary material for: Overexpression of TUBA1A suppresses bladder cancer progression with potential modulation of autophagic activity and macrophage polarization
Source: Front Immunol. 2026 Oct 21;17:1921855. doi: 10.3389/fimmu.2026.1921855 (PMC13730884; doi:10.3389/fimmu.2026.1921855)
Supplement: Supplementary file 1 [file DataSheet1.pdf]

frontiers

The International Society for the Advancement of Cytometry (ISAC) has highlighted the importance of including comprehensive methodological information to ensure data reproducibility and reliability. In line with this, Frontiers in Immunology now requires authors to submit a checklist for manuscripts that involve flow or mass cytometry. This checklist helps standardize the reporting process, improving the quality and transparency of published data. By doing so, we support scientific progress, making it easier for other researchers to replicate and validate experiments.

This form should be submitted with any manuscripts using flow or mass cytometry.

Sample/specimen/material description

☐ Total blood

☐ PBMCs

☐ Organ digests

Other Cultured T24 human bladder cancer cells and THP-1-derived macrophages

Did the samples suffer any treatment before or after incubation with the antibodies?

☒ Drug PMA 100 ng/mL, 24 h; LPS (M1) 100 ng/mL, 24 h; IL-4 (M2) 20 ng/mL, 24 h

☐ Cell permeabilization ____________________

☒ Dye Annexin V used; fluorophore APC and PI

☐ Propidium iodine

☐ Not applicable

Other TUBA1A lentiviral overexpression vs negative control

Instrument and antibodies

Name of the Cytometer hemocytometer

**Antibodies and targets**	**Fluorochrome/ Metal**	**Catalog number/Company**
e.g. anti-CD4	FITC	Cat. XXX/ XXX ltd.
Annexin V-APC/PI (apoptosis probe)	APC/PI	Cat.K2295/APExBIO

Apoptosis counterstain	Unknown	Unknown
anti-IL-12	FITC	Cat.560564/BD Biosciences
anti-CD86	FITC	Cat.374203/BioLegend
anti-Arg1	FITC	Cat.CSB-PA733775HC01MO/ Cusabio
anti-CD206	FITC	Cat.321103/BioLegend

Data analyses

1. Name of the software Flowjo
2. Reference gating strategy in the manuscript or supplementary material

Gating strategy in (eg Figure X) Manuscript Figures 4E, 5E and 5G
